# Time neutrality and reduced ecological burden – handheld robotic-assisted total knee arthroplasty improves operating room efficiency compared with manual total knee arthroplasty

**DOI:** 10.1007/s00264-026-06931-y

**Published:** 2026-07-01

**Authors:** Ricarda Stauss, Hendrik Pott, Peter Savov, Thorsten M. Seyler, Michael P. Ast, Catherine Whittall, Max Ettinger

**Affiliations:** 1 Division of Orthopaedics at Campus Pius-Hospital, Carl von Ossietzky University of Oldenburg, Oldenburg, Germany; 2 Department of Orthopaedic Surgery, Duke University, Durham, USA; 3 Department of Orthopaedic Surgery, Hospital for Special Surgery, New York, USA; 4 Smith & Nephew (United Kingdom), Watford, United Kingdom

**Keywords:** Robotic-assisted total knee arthroplasty, Operating room efficiency, Economic outcomes, Environmental sustainability, Ecological burden

## Abstract

**Purpose:**

To evaluate whether imageless handheld robotic-assisted total knee arthroplasty (rTKA) can achieve time neutrality compared with manual TKA (mTKA) within a standardised workflow, while assessing operating-room (OR) efficiency, environmental impact, and intraoperative workload demand.

**Methods:**

In this prospective study, 24 consecutive patients undergoing primary TKA were stratified to either rTKA (*n* = 12) or mTKA (*n* = 12). A process analysis captured 75 timestamps per case and quantified preparation, surgical, breakdown, turnover, and total OR times. Instrument trays were counted and weighed; reprocessing-related water and electricity consumption and costs were estimated. Perceived workload demand was assessed after both rTKA and mTKA using the NASA Task Load Index.

**Results:**

No statistically significant differences were observed between rTKA and mTKA for any OR time interval (total OR time 79.8 ± 8.1 vs. 75.8 ± 5.3 min, *p* = 0.19; mean surgical time 40.3 ± 4.2 vs. 37.3 ± 3.9 min, *p* = 0.10 for rTKA vs. mtKA respectively). The reduction of one tray for rTKA cases corresponded to estimated savings of €13,900, 3,900 L of water, and 140 kWh of electricity in sterilisation based on an annual volume of 250 cases. For rTKA, the NASA-TLX domain scores demonstrated lower perceived workload and frustration for rTKA than for mTKA (all *p* < 0.001), with a significantly higher perceived performance rating (*p* = 0.025).

**Conclusion:**

Imageless handheld rTKA achieved time neutrality compared with mTKA in a standardised high-volume workflow and was associated with reduced intraoperative workload demand. These findings support the feasibility of integrating handheld robotic assistance without compromising OR efficiency, while potentially improving staff experience and reducing resource utilisation, thereby contributing to value-based arthroplasty care.

## Introduction

Healthcare expenditures in the European Union account for ten percent of the gross domestic product, reflecting the substantial economic burden of modern healthcare systems [1]. Surgical care accounts for approximately one-third of healthcare spending, and the operating room (OR) constitutes one of the most resource-intensive components within the perioperative episode of care [2, 3]. In orthopaedic surgery, total knee arthroplasty (TKA) is among the most frequently performed procedures worldwide, and projections estimate an exponential increase in annual case volume of up to 470% by 2060 [4]. Despite this growing demand, increasing healthcare costs and decreased reimbursement rates pose significant challenges for adult reconstruction programmes [5–7]. Consequently, strategies to decrease cost or maximise OR efficiency while maintaining high quality of care are of critical importance.

Efficiency is defined as the relationship between resources utilised (input) and outcomes achieved (output) and represents a key measure of economic performance [8]. Following an output-oriented approach to optimise OR efficiency, the utilisation of the available resources is modified to achieve the best possible outcome, specifically case volume and OR utilisation [9]. In this context, the most important modifiable factors include the perioperative process times, material and resource utilisation, the OR team, and the implementation of modern technologies [3, 7, 10].

In recent years, the interest and utilisation of robotic-assisted TKA (rTKA) increased substantially. Robotic systems enable improved accuracy and surgical precision, reduced soft tissue trauma and enhanced procedural consistency compared with manual TKA (mTKA) [11–14]. Furthermore, a recent meta-analysis revealed better functional outcomes for rTKA, although these differences did not reach clinically meaningful thresholds [15].

However, the evidence for the impact of robotics on OR efficiency is limited. Concerns persist regarding the initial investment and additional cost per surgery as well as potentially longer surgery duration [16, 17].

Therefore, the purpose of this study was to compare operating room efficiency, environmental impact, and the workload endured by the OR team between rTKA and mTKA procedures.

## Materials and methods

In this prospective, comparative study, a total of 24 patients with end-stage osteoarthritis of the knee underwent either rTKA (*n* = 12) or mTKA (*n* = 12) surgery. Exclusion criteria were defined as bony defects requiring augments and ligament instability requiring a constrained condylar knee (CCK) or hinged TKA.

### Patient population

Demographic and clinical data were retrieved from the digital medical records including age, sex, body mass index (BMI) and American Society of Anaesthesiologists (ASA) score. Pre- and postoperative weight-bearing, anterior–posterior, and long leg radiographs were analysed including the mechanical lateral distal femoral angle (LDFA), medial proximal tibial angle (MPTA), and arithmetic hip-knee-ankle angle (aHKA).

### Surgical technique

All TKA procedures followed a standardised surgical workflow using a standard medial parapatellar approach. A posterior stabilised, fully cemented total knee prosthesis without patellar resurfacing was used in all cases (LEGION™ Total Knee System, Smith & Nephew, Inc., Memphis, USA). In the rTKA cohort, surgery was performed using a handheld, imageless robotic system (CORI™ Surgical System, Smith & Nephew, Inc., Memphis, USA). Surgeries were performed by two senior, high-volume surgeons ([blinded]) with a combined annual caseload of > 800 rTKA procedures. The OR team consisted of six people including the surgeon, two fellows, one scrub nurse and two circulating nurses, and was consistent for all surgical procedures in this evaluation.

### Operating room efficiency

**Fig. 1:**
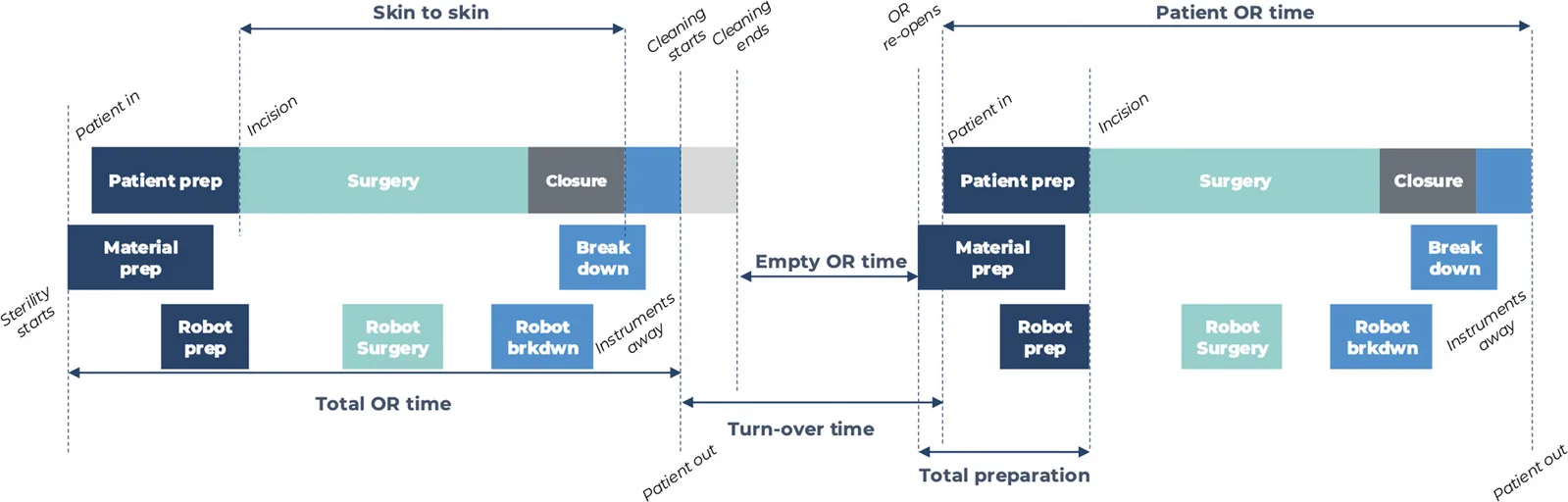
Definition of perioperative procedural times

Process analysis of time-based activities was conducted by breaking down the intraoperative workflow into its individual steps (Fig. 1). Using an AI-enabled camera application (DEO.care, Beringen, Belgium), a medical engineer allocated 75 procedure-specific timestamps to each case using the DEO.care process digital twin platform. Four main intervals were defined: preparation, surgery duration, breakdown, and OR turnover time. Total OR time was defined as the time interval from opening the first sterility pack until the patient left the OR. Patient OR time was less than total OR time, since material preparation started before the patient entered the OR. Surgery duration was defined as skin incision to wound closure and was further subdivided into time intervals including preparation (skin incision to insertion of intramedullary rods (mTKA) or surgical planning (rTKA)), resection, trialling, implantation and wound closure. For rTKA, preparation time was split into the surgical approach, registration of the anatomical landmarks, gap assessment and surgical planning. Breakdown was defined as the time from wound closure to the patient leaving the OR. Turnover was the time from the patient leaving the OR until material preparation started for the next case. Material preparation time, patient preparation time and robot preparation time were monitored, and levels of parallelisation were evaluated while also assessing total preparation time.

Instrument trays were counted and weighed, and reprocessing costs were calculated based on values provided by the institution’s sterilisation company. The environmental impact of the sterilization-related processes was analysed based on the consumption of water and electricity.

### Cognitive and workload demand

Participation in the survey was entirely voluntary, with lead surgeons, trainee surgeons, scrub nurses and circulating nurses invited to complete it after every TKA surgery, for both manual and robotic assisted. The National Aeronautics and Space Administration Task Load Index (NASA-TLX) was used to assess the intraoperative physical and cognitive workload [18]. The survey was hosted on a digital platform (SNAP Surveys, UK) and captured the role of the respondent, the time of day the surgery started (morning, before 12 pm; afternoon, between 12 and 5 pm; evening, after 5 pm), and the respondent’s level of experience with the robotic surgical system (1–4, 5–9, 10–14, 15–20, and more than 20). The NASA-TLX domains were assessed using a bipolar scale from −10 to + 10, with zero representing the neutral midpoint. For the domains mental demand, physical demand, temporal demand, effort, and frustration, negative values reflected lower perceived demand, whereas positive values indicated higher perceived demand. For the performance domain, higher values indicated greater perceived satisfaction with the overall surgical performance. This bipolar scoring approach has previously been applied in robotic arthroplasty workload assessment and facilitates interpretation relative to a neutral reference point [19].

### Statistical analysis

The study was powered based on its primary objective of evaluating operative efficiency and time neutrality between robotic‑assisted and manual instrumentation. Based on prior efficiency analyses conducted by DEO.care, a minimum sample size of ten cases per group was determined to be sufficient to detect clinically meaningful differences in operative time between techniques. NASA‑TLX workload assessments were collected alongside each efficiency‑evaluated case as predefined secondary outcomes and were therefore not independently powered.

Normal distribution was tested using the Shapiro–Wilk test. Chi–square test was used to compare categorical data. Group differences were calculated using Student’s *t*-test or Mann–Whitney U-test. Statistical significance was set at *p* < 0.05. Statistical analysis was performed using SPSS Statistics 29 (IBM Corporation, Armonk, NY, USA) and Microsoft Excel (Microsoft Corporation, Redmond, WA, USA). Figures were created using GraphPad Prism 5 (GraphPad Inc., La Jolla, CA, USA).

The mean NASA‑TLX domain responses were evaluated for each surgical technique. Domain‑specific scores for mental demand, physical demand, temporal demand, effort, frustration, and performance were analysed and compared between groups.

### Ethical approval

The study was approved by the local ethics committee (#2025–244) and was conducted in accordance with the Declaration of Helsinki.

## Results

### Study population

Of 24 patients being enrolled in this study, 18 were female (75.0%). Mean age at the time of the index surgery was 68.5 years. Demographic baseline characteristics are summarised in Table 1.

**Table 1:** Baseline characteristics of the evaluation cohort

	rTKA (*n* = 12)		mTKA (*n* = 12)		*P*-value
Age (y), mean (SD)	68.5	(7.9)	68.6	(8.9)	0.981
Sex, n (%)					0.346
Female	8	(66.7)	10	(83.3)	
Male	4	(33.3)	2	(16.7)	
BMI (kg/m^2^), mean (SD)	33.9	(6.6)	32.3	(3.9)	0.238
ASA score, n (%):					0.025*
1	0	(0.0)	0	(0.0)	
2	6	(50.0)	11	(91.7)	
3	6	(50.0)	1	(8.3)	
4	0	(0.0)	0	(0.0)	
Preoperative MPTA (°)	87.1	(4.1)	88.8	(2.1)	(0.696)
Preoperative LDFA (°)	88.7	(1.4)	88.5	(2.0)	(0.259)

*ASA* American Society of Anaesthesiologists, *BMI* body mass index, *LDFA* lateral distal femoral angle, *MPTA* medial proximal tibial angle, *SD* standard deviation

* Asterisks indicate statistical significance

### Operating room efficiency

**Fig. 2:**
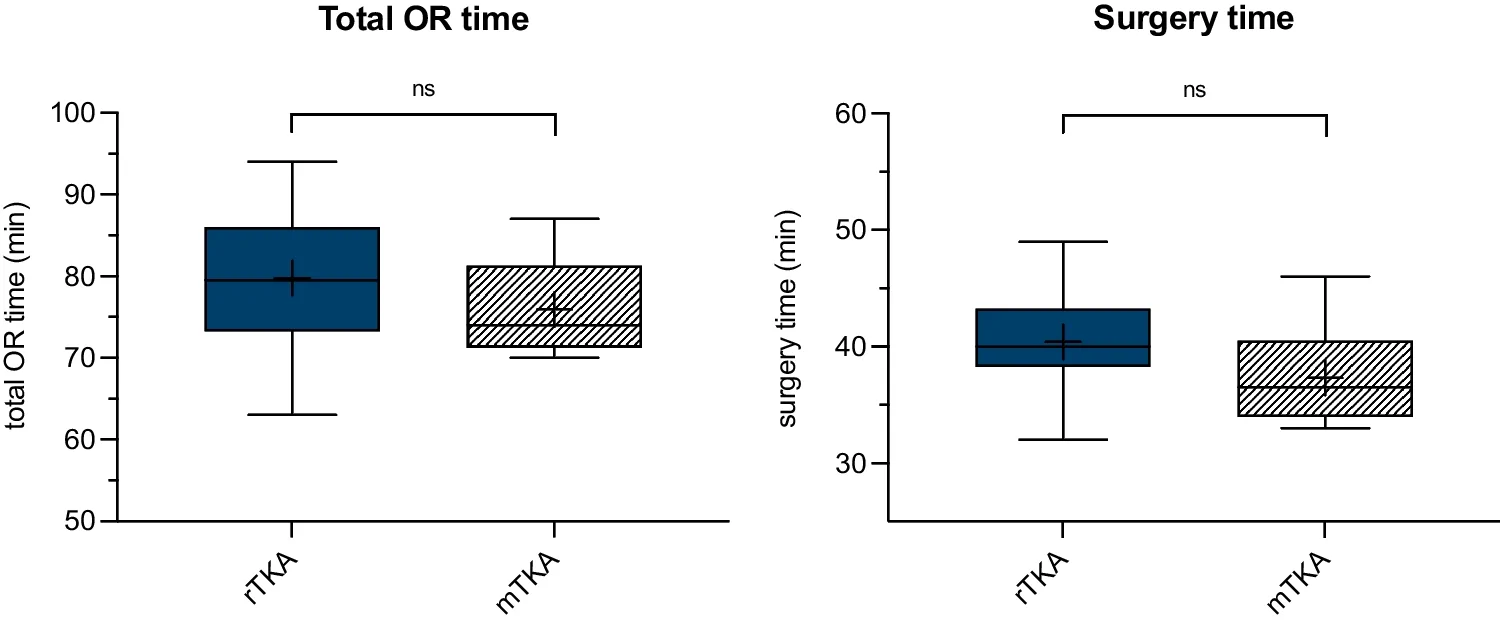
Comparison of total OR time and surgery time between rTKA and mTKA groups

**Fig. 3:**
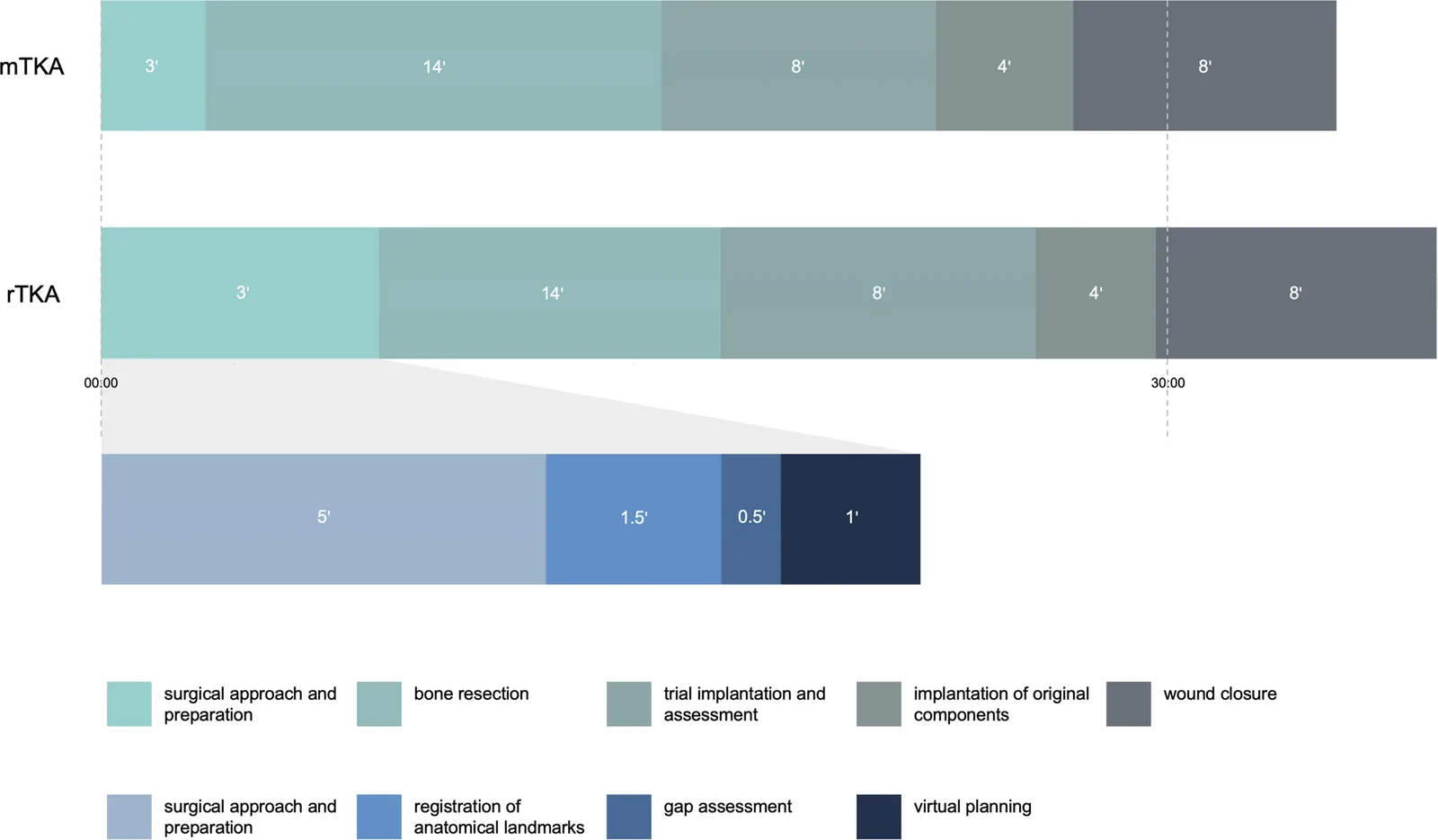
Mean time intervals for mTKA and rTKA. *mTKA* manual total knee arthroplasty, *rTKA* robotic-assisted total knee arthroplasty. *mTKA* manual total knee arthroplasty, *rTKA* robotic-assisted total knee arthroplasty

No statistically significant differences were observed between groups for any time intervals (Table 2). Mean surgical time did not differ significantly between rTKA and mTKA (40.3 vs. 37.3 for rTKA vs. mTKA, *p* = 0.10) (Fig. 2). In the rTKA group, the mean time for the approach was five min, the mean time for the registration of anatomical landmarks and gap assessment was two min and the virtual plan was set in a mean time of one minute, followed by robotic-assisted execution, trialling and cementation of the components. In the mTKA group, the mean time for approach and preparation was three minutes, the mean time for resection was fourteen minutes, trialling was eight minutes, followed by implantation of the final components. The detailed breakdown of surgical steps is presented in Fig. 3.

**Table 2:** Time analysis results

	rTKA (*n* = 12)		mTKA (*n* = 12)		*P*-value
Total OR time	79.8	(8.1)	75.8	(5.3)	0.19
Total patient OR time	62.9	(4.3)	59.1	(5.2)	0.07
Total surgery time	40.3	(4.2)	37.3	(3.9)	0.10
Total preparation time	30.3	(7.3)	29.1	(4.3)	0.63
Patient preparation time	13.5	(2.0)	13.4	(4.0)	0.92
Material preparation time	18.9	(2.3)	19.4	(2.0)	0.61
Total breakdown time	8.8	(1.1)	9.1	(2.4)	0.66
Patient breakdown time	8.8	(1.1)	8.2	(1.1)	0.18
Material breakdown time	10.9	(2.5)	10.9	(5.2)	0.99

Times (in minutes) are presented as means and standard deviation

Analysis of the preparation times spent on the patient, materials and robotic system revealed that all activities were parallelised completely, resulting in time equality for rTKA and mTKA procedures.

### Environmental impact

Both procedures required two generic instrument trays. For mTKA, eight additional procedure-specific trays were used, resulting in a total number of ten trays with a total weight of 50.5 kg. For rTKA procedures, seven procedure-specific trays were utilised, resulting in a total number of nine trays with a weight of 49.1 kg per case.

Based on an annual case volume of 250 TKA procedures, the reduction of one instrument tray in the rTKA group was associated with cost savings of €13,900, a reduction in water consumption of 3,900 L and a decreased electricity consumption of 140 kWh in the sterilisation process.

### TKA workload

**Fig. 4:**
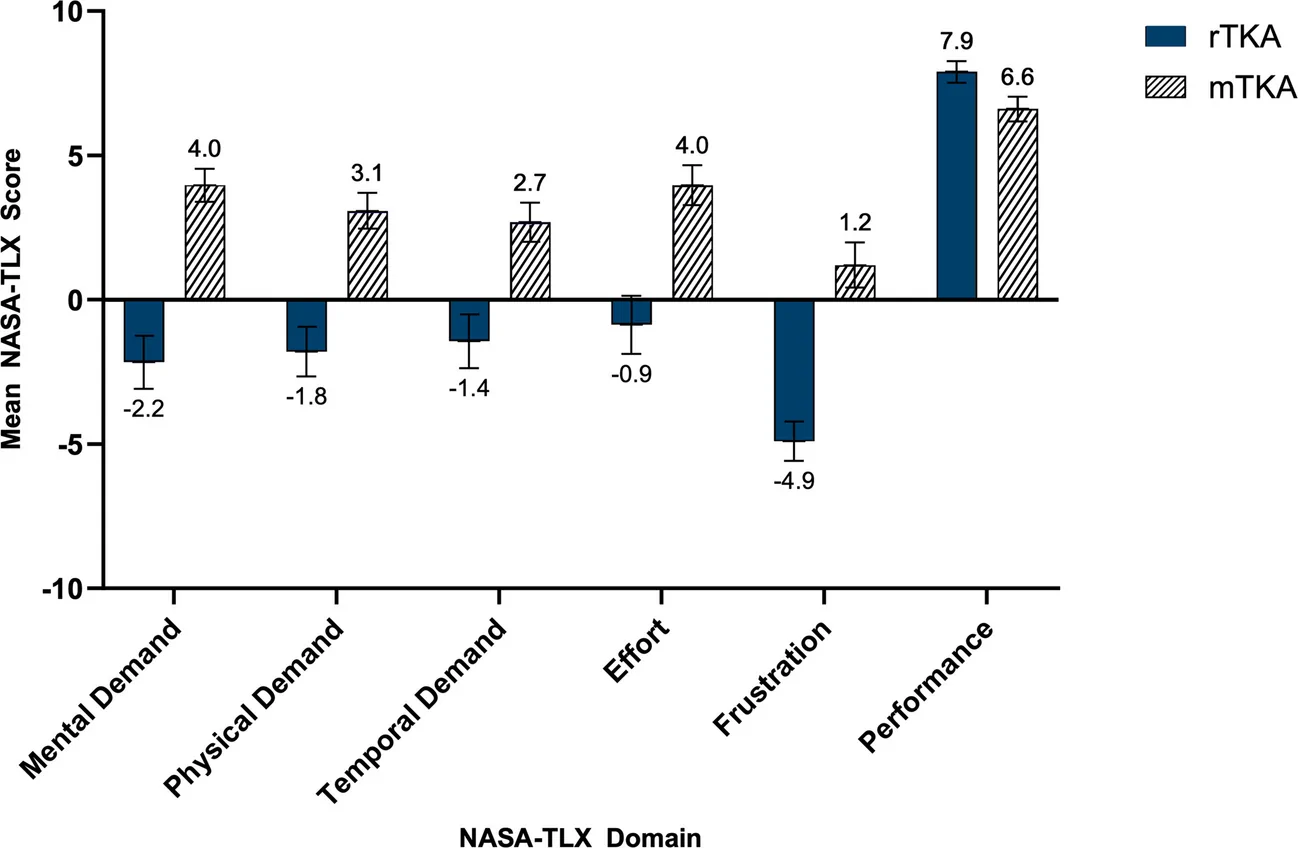
Mean NASA‑TLX domain scores for rTKA and mTKA subgroups. Bars represent mean scores for each NASA‑TLX domain; error bars indicate standard error of the mean (SEM). *NASA‑TLX* National Aeronautics and Space Administration Task Load Index*, mTKA* manual total knee arthroplasty, *rTKA* robotic-assisted total knee arthroplasty

A total of 56 NASA-TLX questionnaires were completed across the 24 procedures (30 after rTKA and 26 after mTKA), reflecting independent assessments by individual operating-team members rather than the number of patients. All NASA-TLX domains were significantly lower in rTKA compared to conventional mTKA procedures (Table 3, Fig. 4). Surgeons were significantly less mentally strained (−2.17 vs. 3.96, *P* < 0.001), less physically exhausted (−1.80 vs. 3.01, *P* < 0.001), experienced less time pressure (−1.43 vs. 2.69, P < 0.001), required less effort (−0.87 vs. 3.96, *P* < 0.001), and were more satisfied (less frustrated) with the surgical procedure (−4.90 vs. 1.19, *P* < 0.001). Overall, the OR team was more likely to be satisfied with the final performance (7.90 vs. 6.62, *P* = 0.025).

**Table 3:** NASA‑TLX domain scores for rTKA and mTKA subgroups

	rTKA (*n* = 30)		mTKA (*n* = 26)		*P*-value
**NASA-TLX domains**					
Mental demand	−2.17	(5.02)	3.96	(2.96)	< 0.001 *
Physical demand	−1.80	(4.69)	3.08	(3.16)	< 0.001 *
Temporal demand	−1.43	(5.13)	2.69	(3.44)	< 0.001 *
Effort	−0.87	(5.53)	3.96	(3.53)	< 0.001 *
Frustration	−4.90	(3.72)	1.19	(4.03)	< 0.001 *
Performance	7.90	(2.02)	6.62	(2.14)	0.025 *

Values are presented as mean (standard deviation) on a bipolar scale ranging from −10 to + 10, with zero representing the neutral midpoint. For the domains mental demand, physical demand, temporal demand, effort and frustration, lower values indicate lower perceived workload. For the performance domain, higher values indicate greater perceived satisfaction. P‑values indicate between‑group comparisons for each NASA‑TLX domain. * Asterisks indicate statistical significance

*NASA‑TLX* National Aeronautics and Space Administration Task Load Index, *mTKA* manual total knee arthroplasty, *rTKA* robotic-assisted total knee arthroplasty

## Discussion

The most important finding of the present evaluation is that primary rTKA performed with an imageless handheld system achieved time neutrality when compared with conventional mTKA. Despite concerns that robotic adoption may prolong operative workflows, we observed no statistically significant differences in total OR time, patient OR time, preparation, or breakdown intervals between groups. In parallel, rTKA reduced the environmental burden by decreasing instrument tray utilisation with consecutive reductions in sterilization-related resource consumption. Furthermore, it was associated with substantially lower perceived workload demand across all NASA-TLX domains for the operating team. Taken together, these findings suggest that, in an output-oriented framework of OR performance (i.e., maximising throughput and quality while minimising resource consumption), imageless handheld rTKA can support operational excellence without sacrificing efficiency [8, 9, 17].

### Time neutrality and OR efficiency

OR time is a key driver of perioperative cost and capacity, and the OR remains one of the most resource-intensive components of surgical care [2, 3]. While robotic systems have been associated with improved precision and consistency [11–15], their widespread implementation is often challenged by perceptions of longer operative time, added staffing requirements, and disruptions to established workflows [16, 17]. In our cohort, the detailed time-stamp analysis did not demonstrate prolongation of any major OR interval for rTKA. Although rTKA introduces additional steps (e.g., landmark registration and intraoperative planning), these were offset by efficiencies in subsequent phases, resulting in comparable overall surgical time. These findings align with the concept that efficiency gains are achievable through standardisation and high team proficiency, particularly in high-volume arthroplasty settings (7, 10). This observation is consistent with published evidence indicating that operative time associated with robotic‑assisted TKA is highly dependent on workflow design and team experience rather than robotic assistance itself [17, 20, 21].

An important determinant of operating room efficiency in rTKA is the learning curve associated with the adoption of the technology. Previous studies have reported a learning curve of seven to 11 cases to reach the inflexion point towards the proficiency phase for surgical time, but no learning curve has been observed for the accuracy of component positioning or the precision of bone resection [22–24]. While some studies have reported complete normalisation of the operative time relative to manual TKA for high-volume surgeons [25], others revealed significantly longer operative times for robotic procedures [24]. These differences suggest that workflow efficiency is strongly influenced by surgeon experience, team proficiency, and procedural standardisation. In this present study, time neutrality was achieved for rTKA, which may be attributable to the highly standardized operative environment, the extensive robotic experience of the participating surgeons and the presence of a dedicated OR team proficient in the robotic workflow.

A further explanation for time neutrality may relate to the characteristics of the robotic platform evaluated. Imageless systems avoid preoperative CT acquisition and associated logistical steps, which can otherwise affect scheduling and resource use. In addition, a handheld, mobile device with a small footprint may facilitate room set-up and reduce constraints in confined OR environments. These workflow characteristics may be particularly relevant when aiming to preserve OR utilisation and throughput, an outcome increasingly emphasised in performance benchmark studies for hip and knee arthroplasty [7] and in broader discussions of workflow efficiency with robotic arthroplasty [17].

Medical case complexity, reflected by higher ASA classes, is associated with greater perioperative complexity and therefore may influence patient preparation, anaesthesia management and overall operating room metrics. Notably, the rTKA cohort included a significantly higher proportion of ASA III patients than the mTKA cohort. Despite this baseline imbalance, no statistically significant group differences were observed in patient preparation time, surgery duration, patient OR time and total OR time. These findings suggest that robotic assistance can be integrated into a standardized surgical workflow without compromising operating room efficiency, despite a greater burden of systemic comorbidity in the rTKA cohort. This observation is consistent with evidence that robotic assistance can reduce the surgery duration, length of stay, and 90-day complication and readmission rates of complex TKA to the level of noncomplex procedures [26]. However, the present study was not designed to evaluate the influence of medical case complexity on operating room efficiency, and future sufficiently powered studies are warranted to further investigate this relationship.

### Environmental impact and resource utilisation

Environmental sustainability is emerging as an additional dimension of value-based care in orthopaedics. Surgical services account for a substantial proportion of healthcare resource consumption, and procedure-specific consumables and reprocessing are important contributors. In the present evaluation, rTKA was associated with a reduction of one procedure-specific tray compared with mTKA, translating into measurable reductions in reprocessing-related cost as well as water and electricity consumption at the institutional level. Although the absolute differences per case are modest, cumulative effects become meaningful at typical arthroplasty volumes, supporting the concept that workflow and instrument rationalisation can contribute to reducing the ecological burden of surgical care. In this context, life‑cycle analyses have demonstrated that reprocessing and sterilisation of reusable instruments account for a substantial proportion of the procedural carbon footprint for total knee arthroplasty. McGain et al. reported that energy used to disinfect and steam‑sterilise reusable equipment contributed approximately 43.4 kg carbon dioxide equivalent (CO₂e) per TKA, representing roughly one third of the surgical emissions [27]. Similarly, Rizan et al. demonstrated that sterilisation and reprocessing contribute approximately 20–30% of procedural emissions across common surgical procedures [28]. These findings support the potential environmental benefit of instrument rationalisation strategies in arthroplasty programmes with consecutive reduced sterilization-related resource utilization.

Importantly, instrument optimisation does not necessarily conflict with safety or standardisation; rather, it can be supported by structured process redesign. Recent work has highlighted that applying manufacturing efficiency principles can improve OR efficiency in rTKA [20]. From a health-economic standpoint, reduced instrument burden may also partially offset the incremental costs associated with robotic technology [16, 17], particularly when considered alongside potential clinical benefits reported for rTKA such as improved radiographic accuracy and reduced soft-tissue trauma [11, 13, 15]. Further studies that integrate full life cycle and cost analyses are warranted to quantify these trade-offs more comprehensively.

### Team wellbeing and cognitive workload

Beyond time-based metrics, our results demonstrate significantly lower perceived workload during rTKA across all NASA-TLX domains for both surgeons and scrub nurses. Cognitive workload has been linked to technical performance, decision-making quality, and team communication, and validated measurement tools are increasingly used to evaluate intraoperative demands [18]. The observed improvements in mental, physical, and temporal demand as well as reduced frustration may reflect greater procedural consistency and decision support during bone preparation and balancing, which may be particularly valuable in complex cases and during longer operating lists. These findings are consistent with emerging evidence suggesting that perceived workload during robotic arthroplasty improves with experience and workflow familiarity, and that reductions in physical demand and frustration may persist beyond the initial learning phase.

Previous studies using NASA‑TLX have demonstrated its validity and reliability for assessing intraoperative workload in robotic surgery and highlight the influence of team experience rather than technology alone on perceived cognitive demand [18, 19, 29].

From an operational perspective, reduced team strain may have downstream relevance for sustaining efficiency and quality in high-demand arthroplasty programmes. While the present study was not designed to assess burnout, absenteeism, or staff turnover, our findings suggest that the ergonomic and cognitive aspects of robotic workflows deserve consideration when evaluating the overall value proposition of robotic arthroplasty, alongside classic endpoints such as radiographic accuracy, functional outcomes, and costs [11–17].

Of note, medical case complexity represents a potential confounding factor that may influence the perioperative workflow, intraoperative demands, and team perception of workload. In this present study, medical case complexity reflected by ASA classification was higher in the rTKA group, whereas NASA-TLX domain scores demonstrated a lower perceived workload for rTKA cases. This observation may be attributable to the highly standardized robotic workflow and procedural familiarity within a dedicated team. Although there were no group differences for the procedural anaesthesia times, higher case complexity might influence the anaesthesiologists’ perception of workload and respective subscales which have not been assessed in this present study.

## Limitations

The results of this evaluation should be interpreted considering several limitations. This was a single-centre prospective case–control evaluation with a limited sample size, which reduces statistical power to detect small differences in time-based outcomes and increases the risk of type I and type II errors. Therefore, the results should be interpreted as preliminary workflow and feasibility data. Furthermore, procedures were performed by two senior high-volume surgeons using one imageless handheld robotic platform; therefore, results may not generalise to other institutions, operating room team compositions, surgeon experience levels, case mixes, or robotic systems including image-based platforms. Larger multicentre studies are warranted to investigate whether time neutrality can be consistently achieved across different practice settings and levels of robotic experience. Although we quantified reprocessing-related resource use (tray number/weight and associated water and electricity consumption), we did not perform a comprehensive cost-effectiveness analysis incorporating acquisition, maintenance, disposables, or broader life-cycle environmental impacts. Therefore, comprehensive life-cycle analyses are required to evaluate the environmental impact and carbon footprint of rTKA. Notably, robotic-assisted TKA may carry a higher overall carbon footprint than manual TKA once manufacturing and capital-equipment life cycles are considered, even where instrument-tray reduction lowers reprocessing-related emissions [30]. Another important limitation is the inherent subjectivity of the NASA-TLX assessment, consequently, the NASA-TLX is susceptible to recall bias. Furthermore, the surgical team could not be blinded to the procedure type; consequently, perceived workload may have been influenced by institutional preference, familiarity with the robotic platform, or enthusiasm bias towards modern technologies. The results should therefore be interpreted as perceived workload in an experienced robotic centre. Lastly, the learning curve was not formally assessed in this evaluation; however, both surgeons had performed more than 500 rTKA procedures prior to enrolment, placing them well beyond published proficiency thresholds for robotic-assisted TKA, which a recent systematic review and meta-analysis pooled at a median of 17 cases (interquartile range 9–27) [31].

## Conclusion

In conclusion, imageless handheld rTKA achieved time neutrality compared with mTKA while reducing environmental impact and lowering perceived intraoperative workload. These findings support the technical and operational feasibility of integrating robotic surgical systems into high-volume arthroplasty workflows without compromising OR efficiency, whilst highlighting additional value dimensions, including resource stewardship and team wellbeing, that may be relevant to value-based evaluations of robotic arthroplasty. Future studies, including multi-centre randomised controlled trials with formal cost-effectiveness analyses, are warranted to confirm the results of this preliminary workflow analysis and to further characterise the ecological and economic impact of rTKA using standardised, procedure-level life cycle assessment methods.

## Data Availability

The data that support the findings of this study are available on request from the corresponding author.
